# Evidence-Based Laboratory Medicine for Cardiovascular Disease Management in Mental Healthcare: A Literature Review

**DOI:** 10.7759/cureus.108928

**Published:** 2026-05-15

**Authors:** Bupe A Kyelu, Phillip T Bwititi, Sita Sharma, Ezekiel U Nwose

**Affiliations:** 1 School of Health, Psychology and Medical Sciences, Royal Darwin Hospital, University of Southern Queensland, Toowoomba, AUS; 2 Dentistry and Medical Sciences, Charles Sturt University, New South Wales, AUS; 3 School of Nursing and Midwifery, University of Southern Queensland, Ipswich, AUS; 4 School of Health and Medical Sciences, University of Southern Queensland, Toowoomba, AUS

**Keywords:** cardiovascular complications, estimated whole blood viscosity (ewbv), evidence-based laboratory medicine (eblm), mental healthcare, pathology test

## Abstract

Cardiovascular disease (CVD) comorbidity in mental health underpins the basis of referrals to cardiology specialists in mental healthcare, while blood flow issues constitute part of the pathophysiology. However, there is a dearth of data on evidence-based cardiovascular medicine in mental healthcare, such as clinical laboratory testing of whole blood viscosity (WBV). The primary objective of this literature review is to assess the level of evidence-based laboratory medicine (EBLM) practice for CVD management in mental healthcare, including aspects of demography, CVD complications, guidelines for management, and laboratory monitoring with WBV. This study followed the Preferred Reporting Items for Systematic Reviews and Meta-Analyses extension for Scoping Reviews approach. Four types of evidence constituted the phenomena of interest, which included demography, epidemiology, CVD, guidelines, and pathology. Two databases, including Our World in Data and PubMed, were targeted for search, resulting in empirical and non-empirical data. The inclusion criteria for the literature were the indication of data on “CVD and mental health.” The search was done in four separate searches to cater to each phenomenon of interest with associated keywords. Further, grey literature was also sought from other sources, including, but not limited to, Google Scholar. Anxiety and depression constitute about 89.36% of the burden of mental health. In terms of individual disease prevalence, anxiety and depression combined is 11.10%, followed by alcohol and other drugs at 3.6% of cases in Australia. Prevalence was more among young and mid adults (50% out of 76%), unlike the global levels (39% out of 69%). Guidelines were missing in the literature, with nurses and support management having little mention. Blood viscosity has been a well-established research topic, but not in mental healthcare. The review identifies gaps in knowledge and practice of EBLM for CVD in mental healthcare. From a mental health nursing perspective, the higher prevalence among younger adults in Australia is unexpected, which perhaps calls for case studies for early identification and intervention against potential CVD complications in young people. The significance for further studies lies in EBLM cardiovascular preventive clinical practice.

## Introduction and background

Cardiovascular disease (CVD) complications include blood flow pathophysiologies such as venous thrombosis and peripheral artery disease among mental healthcare clients [1]. In clinical laboratory medicine, the pathophysiology of blood flow can be confounded by any of three CVD factors, collectively known as Virchow’s triad: hypercoagulability, endothelial dysfunction, and stasis. Any of these three factors can cause or confound CVD. Conversely, management of any of the three factors is part of the treatment regimen. For instance, it has long been indicated that aspirin and pentoxyflline improve blood flow by reducing whole blood viscosity (WBV) in heart disease [2].

WBV is a test that detects the level of stasis, i.e., the thinness or thickness of the blood, and informs on the effects of its flow or pooling [2-4]. Blood viscosity is also a factor in various diseases involving CVD complications. Clinical laboratory tests for WBV can be used to assess the efficacy of blood-thinning medications [2,5]. However, the affordances (i.e., affordability and availability) constitute a limiting barrier for the test. Therefore, algorithms for estimated whole blood viscosity (eWBV) are being investigated as a cheap and universally available option [6].

Indeed, in mental health management, the eWBV method has been suggested for over 25 years [7]. Yet, it is pertinent to highlight that eWBV can be derived/calculated from hematocrit and serum protein results, which are routine and affordable pathology tests [8]. This implies there is a gap in knowledge and practice to explore.

Considering that alcohol and other drugs (AODs) and individuals on antiplatelet management may dominate the subpopulation of patients attending mental health facilities, laboratory monitoring of these clinical cases has yet to become a part of practice. It suffices that while improving evidence-based laboratory medicine (EBLM) for CVD in mental healthcare is a primary focus, the related factors constitute themes of interest. Especially, the epidemiological data or global statistics linking CVD and severe mental illness are of interest in this review to substantiate empirical evidence of the need for the EBLM.

Objective

This review aims to explore globally and in Australia the phenomenon of EBLM practices for the management of blood flow issues associated with CVD in mental healthcare services.

Review questions

This study has four research questions, including one for each theme: (1) What is the demography of CVD among mental health clients? (2) What is the prevalence of CVD in mental healthcare? (3) What are the guidelines for management in mental healthcare? (4) What are the laboratory monitoring tests for blood flow issues?

Study focus

The first and second questions about demography and prevalence constitute a “prelude of empirical data” to provide justification of concern. The third question seeks to determine the existence of policies and procedures that clinicians are required to comply with for quality evidence-based medicine practice. Specifically, this third question seeks to determine the existence of EBLM guidelines in mental health practice. The fourth question is the crux of the matter, i.e., it extends on the preceding third question to focus EBLM on laboratory monitoring tests for blood flow issues.

## Review

Methodology

Study Design

This literature review may be viewed as narrative but was designed to follow the scoping Preferred Reporting Items for Systematic Reviews and Meta-Analyses extension for Scoping Reviews (PRISMA-ScR) method, with literature searches performed discretionally on two databases, including PubMed and Our World in Data (OWiD). A grey literature search was also performed using the Google platform. The literature search was done by two of the coauthors, including stages of identification through to final selection/inclusion.

Search Protocol

The search process and other details, including review appraisals, were as published/registered in the review protocol. The details in the published protocol include the review process and how our separate scoping reviews were performed, as well as data extractions and analysis, among others [9].

Search Strategy

The PRISMA-ScR search strategy involved an initial iterative development, i.e., repeated searches on multiple platforms, whereby it was established that using the Boolean operator “AND” to combine all the four phenomena of interest (demography of CVD in mental health, epidemiology of CVD in mental health, guidelines for EBLM management of CVD, and laboratory monitoring tests for blood flow issues in mental healthcare) was yielding inferior search outcomes compared to performing separate PRISMA-ScR for the different phenomena. Therefore, discretion was applied to perform four separate thematic searches using one core indexed database and afterwards supplemented with another indexed database (Google) for grey literature, as well as a non-indexed database (OWiD) for secondary quantitative empirical data.

The iterative development for the PRISMA-ScR of the four separate three-step processes leading to inclusion revolved around starting with a search for identification that was initially unlimited to date of publication or article type, followed by limitation to records with available free full texts. Screening was by title matching the phenomenon of interest, including demographic terms, epidemiologic terms, guidelines, and pathology terms. For the demography of CVD in mental health, epidemiology of CVD in mental health, and guidelines for EBLM management of CVD in mental healthcare, the Boolean operator “AND” was used to add meta-analysis for the demographic as well as the epidemiological search. For the laboratory monitoring tests for blood flow issues in mental healthcare (i.e., the focus of work), screening utilized the Boolean operator “OR” via title matching, i.e., “laboratory medicine” OR “pathology.”

Eligibilities were limited to the last one year for the demography of CVD in mental health, lack of data for the epidemiological theme, and limitation to <20 years and availability of data for the guidelines piece. For the laboratory monitoring tests for blood flow issues in mental healthcare, eligibility was determined by blood viscosity, laboratory, or pathology tests.

It is pertinent to emphasize that researchers were cognizant that searching two or more databases maximizes the yield or search outcomes. Hence, it was determined that separating the themes of this broad topic and performing separate PRISMA-ScR on one core primary database (PubMed) was superior to one search process on multiple databases (PubMed and Google). Of note, Google and OWiD were added; hence, the flexibility that is permissible in scoping review has been adopted in using multiple platforms to provide depth and nuance.

Analysis

This study adopted mixed-method analysis, including qualitative and quantitative methods. For the qualitative data, visual analysis of “word cloud” and annotated bibliography were adopted, while descriptive frequency was used for the quantitative data.

For the literature selected from PubMed, critical appraisals were performed using a standard seven-item questionnaire adopted from published literature [10]. A minimum of two and up to four public health scholars were invited to appraise each article using the tabulated checklist of seven questions [9]. Three out of seven questions were used for qualitative analysis, while four out of seven questions were adopted for categorical (i.e., quantitative) analysis. The three qualitative questions were “What do you think are the main strengths and weaknesses of the study?”; “How do these results fit into the wider evidence base?” and “What are the public health and policy implications of the findings?”

The qualitative analyses were done twice. First, the Word Cloud tool was used to analyze the opinions of independent reviewers. This method was adopted as a valid tool to qualitatively analyze the perspectives of clients [11]. In this study, cognizance was made that the method suits nursing and public health practices, whereby nurses and public healthcare practitioners listen to clients and analyze their words to identify issues. Word clouds were developed from the reviewers’ responses and visually analyzed for key words, i.e., text sizes. Second, there was the evaluation of the annotated bibliography for keywords as per the phenomenon of interest.

Quantitative analysis was also performed twice. First, four out of seven categorical review questions were quantitatively analyzed as a cumulative assessment of the quality of articles with regard to adoptability in clinical practice, and this was determined by the number of “Yes” responses to each of the four out of seven questions.

For the empirical data from OWiD, analysis was by simple descriptive frequencies. This study sought to review four themes that make up the broad phenomenon of interest; hence, the adopted review methods and heterogeneity of the themes precluded a meta-analysis and other common statistical measures.

Grey literature was also searched to provide nuance to the discussion. The search platform included, but was not limited to, Google Scholar, as well as following up on publication titles of interest in the reference list. This grey search method was used in generating evidence of applicable uses of WBV.

Results

Critical Appraisal Findings

**Figure 1:**
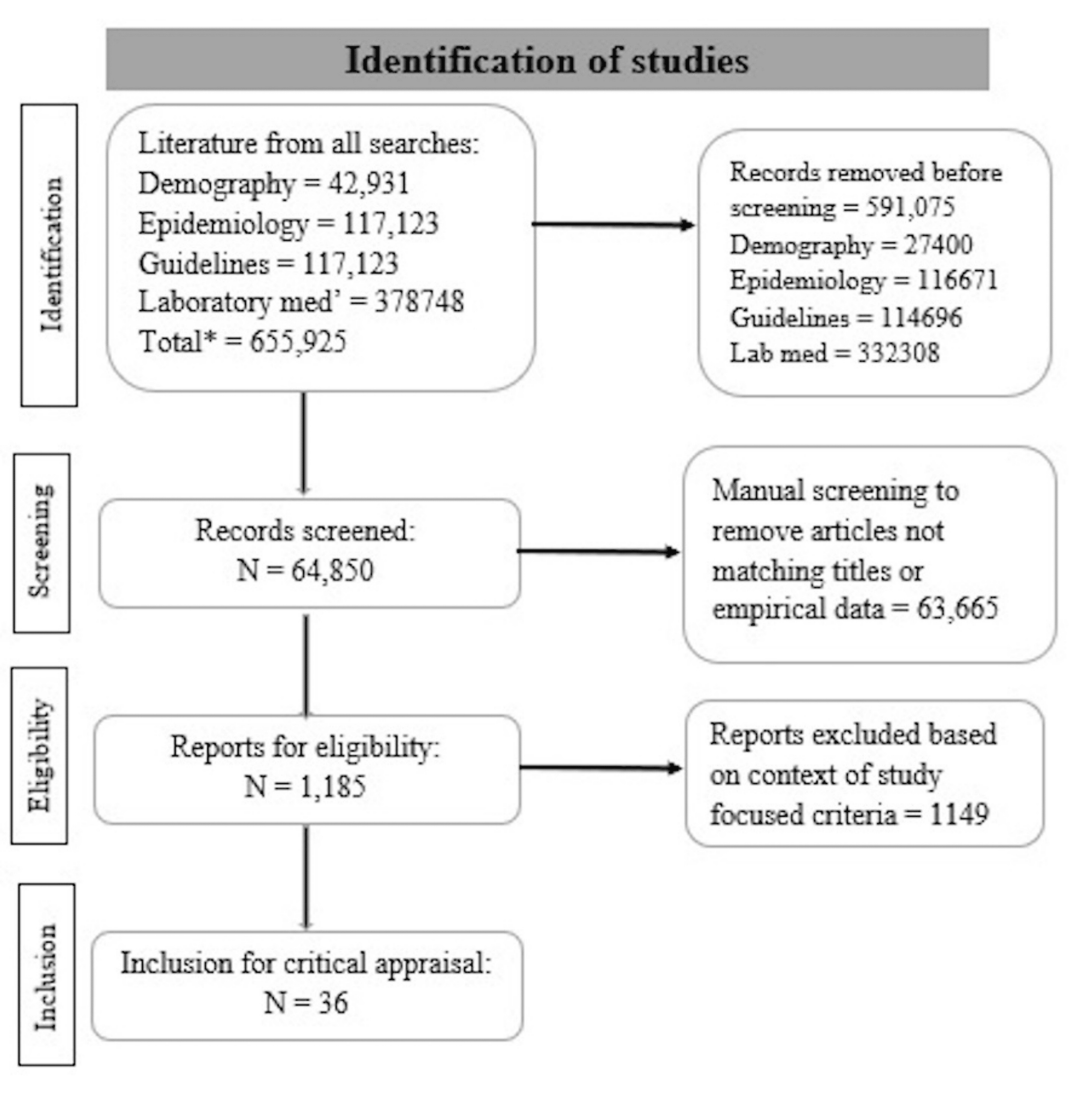
Preferred Reporting Items for Systematic Reviews and Meta-Analyses extension for Scoping Reviews (PRISMA-ScR) flowchart.

Figure 1 presents a summary of the literature search outcome, viz, a combination of four search outcomes, i.e., four PRISMA flowcharts summed. The distribution of the 36 articles into the four separate searches is tabulated into the various research questions, matching the various themes.

Distribution of the 36 included articles is separated according to the different themes’ search. Demography of CVD among mental health clients yielded 10 articles [12-21]. Prevalence of CVD in mental healthcare yielded eight articles [22-29]. Guidelines for management in mental healthcare yielded 10 articles [30-39], and the laboratory monitoring tests for blood flow issues resulted in eight articles [40-47].

Qualitative Analysis

Table 1 provides a summary of both the annotated bibliography and the Word Cloud. Further points of outcome for each thematic review are discussed below.

**Table 1:** A summary of qualitative reviews. PTSD = post-traumatic stress disorder; CVD = cardiovascular disease

Theme	Analysis	Outcome
Demography	Word cloud	Gender and marital status are the most common specifications among demographic variables. PTSD is the most common among comorbidity determinants
	Annotated bibliography	Demography was a focus in 2/10 (20%). One of the two articles considered age, gender, and marital status [20]. Another article discussed the educational status of clients [19]. Anxiety and depression were mentioned 22 and 23 times, respectively, which reflects the focus of health conditions in discourse
Epidemiology of CVD in mental health	Word cloud	COVID-19, diabetes, and depression are common epidemiological iterations
	Annotated bibliography	Depression, diabetes, and PTSD. Out of the 2,958-word bibliography (excluding iterations in title/heading), CVD is indicated 17 times, mental health is mentioned 23 times, and depression is mentioned 4 times
Guidelines	Word cloud	Guidelines appear to be missing in the reviewers’ summaries. However, 6 relevant words that can form a statement are studies improving mental health systematic evaluations. The other 10 words are coverage-reported disorders focusing on anxiety/depression among young people. Another notable abbreviation is PDSA, which stands for Plan-Do-Study-Act
	Annotated bibliography	Out of the bibliography of 3,239 words, “guide” is mentioned twice, but the two words include one in the heading and the other referencing guidelines of the adopted research method. However, there is implicit PDSA, which showed two counts
Pathology evidence base	Word cloud	5 relevant words that could make a sentence are improving mental health systematic evaluations. Other 6 words are anxiety/depression, disorders, estimates, prevalence, and young people. The orogram evaluation approach is yet another phrase that could be coined
	Annotated bibliography	Frequency counts of “blood,” “pathology/pathological,” “stasis,” “viscosity,” and “evidence” in “mental” health were mentioned 19 times, collectively. Blood flow is mentioned thrice, including the indication of benefit in monitoring the risk of CVD and stress-induced pathophysiology [43], as well as the usefulness of blood viscosity in monitoring microvascular blood flow and antiplatelet therapy [47]. There is also a reference to assessing blood flow among individuals with metabolic syndrome [46]

Demographics of CVD among mental health clients: Word Cloud analysis showed that, besides post-traumatic stress disorder (PTSD), other applicable diseases indicated included epilepsy and related symptoms. EBLM is also implied to be relevant for treatment outcomes. Perspectives are clear on policy implications for intervention/treatment, service improvement, health promotion, individuality in support services, and research.

Prevalence of CVD in mental healthcare clients: Word Cloud analysis showed that the strengths of the articles lie in emphasis on data and diagnostic approaches, as well as criteria and guidelines. Analysis of the annotated bibliographies of CVD showed that risk is mentioned 20 times, which included associated mortality, risk factors, risk of bias, risk of being female, risks of long-term complications, poor health, diabetes with antipsychotics, perinatal mental health, and depression.

Guideline articles: Word Cloud analysis showed that, regarding policy implications, relevant words that included improving help-seeking programs, promoting service outcomes, and quality could be coined. Nurses and support management were little mentioned but noteworthy.

Analysis of the annotated bibliography showed that one of the two articles mentioned Plan-Do-Study-Act (PDSA) advanced challenges of “absence of a long-term strategic plan, resistance to change … and the technical experience of staff” [33]. The second article investigated PDSA methods in the context of quality-of-care improvement strategies and reported 4% adherence to key methodological principles: “that there is a continued need for improvement in quality improvement methodology” [31].

Evidence: Eight iterations in the content; among these, four notable points are described in the following paragraphs:

Paper on “Bridging the science-practice gap in … mental health” identified that “although scientists often focus on generating evidence-based innovations, end-users apply their own criteria to determine an innovation's value”; and also that “developers of models of care in aging, dementia, and mental health typically fail to incorporate the complexities of health systems,” including the barriers to integration [34].

Another paper bordered on guidelines for the “referral process from primary to specialised mental health care.” Four indices were recommended from 16 preliminary indicators [35], but neither CVD nor pathology was mentioned.

The article that focused on guideline-related study was on video consulting, with the key findings of “significant lack of evidence” and “a more definitive evidence base is urgently needed to support extending use” [33].

Likely, the most relevant to the phenomenon of interest was the article on “association of data integration technologies with intensive care clinician performance.” The authors investigated published evidence on the association of user-centered data integration and visualization technologies (DIVTs) with clinician performance. A key finding was that “any DIVT was an improvement over paper-based data in terms of self-reported performance, mental and temporal demand, and effort,” but significant “work remains to identify which visualizations and technologies” are effective. Further, the authors recommend “standardizing human factors testing by developing a repository of open access benchmarked test protocols, using a set of outcome measures, scenarios, and data sets, may accelerate the design and selection of the most appropriate DIVT” [30].

Pathology articles: Word Cloud analysis showed that laboratory testing is indicated as a phenomenon of interest. Regarding evidence base, data, measures, and technology are little mentioned but noteworthy. Analysis of the annotated bibliography highlights blood viscosity as a common CVD symptom and evidence-based effect of treatment in reducing hyperviscosity syndrome [41], including assessing antiplatelet therapy on lipidemia in patients with CVD [40], and the impact of aerobic exercise on CVD [42]. Further, one study [44] acknowledged that “rheology parameter could bring additional prognostic information for the management … Most medical centres are not equipped to measure this parameter properly.” The authors suggested ultrasound imaging as an alternative [44], yet cognizance must be given to the affordances (i.e., accessibility and affordability) of ultrasound services that are still being improved upon [48].

Quantitative Analysis

Depression and anxiety contribute 89.36% of the estimated yearly prevalence of mental illnesses. In the burden of non-communicable diseases, mental disorders, and CVD contributed approximately 155 million and 428 million, respectively, in 2021. For the same period, data from Australia contributed 739,042 mental disorders and 828,043 CVD cases. CVD ranked highest in the burden of disease causes. However, there is limited work on epidemiology and evidence. WBV is indicated in concepts but not among the empirical data evaluated.

Critical appraisal of literature articles: Based on the standard appraisal tool [10], quantitative assessments of basic questions are presented in Table 2. For the guideline and pathology themes, the percentage of affirmative (Yes) responses by reviewers to all questions was 100%.

**Table 2:** Percentage of affirmative (yes) responses by reviewers. CVD = cardiovascular disease

	Question	Percentage
Demographics of mental health clients	Is the study type appropriate for the research question?	91.67%
	Is the sample representative of the population being studied?	66.67%
	Can you generalize from the population being studied?	81.33%
	What are the main results and are they presented in an understandable way?	91.67%
Epidemiology of CVD among mental health clients	Is the study type appropriate for the research question?	100.00%
	Is the sample representative of the population being studied?	57.14%
	Can you generalize from the population being studied?	85.71%
	What are the main results, and are they presented in an understandable way?	100.00%

Demographic data on mental health is limited to the categories and the contribution of each category to the burden of disease. A cursory analysis showed that all mental health categories contributed 9.4% to the global burden of disease. Critical analyses showed a few findings. Table 3 shows the contributions of the prevalence of each mental health category to the global burden of mental illness. Table 4 shows the prevalence of mental health problems by age and gender. It highlights women constituting the majority, as well as being consistently higher in Australian stratified age groups relative to the global levels. Further, the prevalence of the various mental illnesses is presented in Table 5.

**Table 3:** Distribution of categorized disorders in the global burden of mental health.

Disorders	Proportion
Depression	46.81%
Anxiety	42.55%
Bipolar	5.32%
Schizophrenia	3.19%
Eating	2.13%
Total	100.00%

**Table 4:** Proportion of mental health by age and gender in Australia and globally in 2021.

Variable	Group	Australia	World
Mental health in gender	Males	4.00%	3.20%
	Females	5.80%	4.80%
Depression in stratified age groups (years)	5–14	1.37%	0.69%
	15–19	6.22%	3.38%
	20–24	8.17%	4.68%
	25–29	7.73%	4.98%
	30–34	7.34%	5.19%
	35–39	7.31%	5.73%
	40–44	7.06%	6.12%
	45–49	6.45%	6.23%
	50–54	5.81%	6.31%
	55–59	5.15%	6.42%
	60–64	4.66%	6.45%
	65–69	4.41%	6.38%
	70+	4.06%	5.94%

**Table 5:** Prevalence of mental health in Australia relative to the global level.

Disorders	Australia	World
Anxiety disorders	6.20%	4.40%
Depressive disorders	4.90%	4.00%
Drug use disorders	1.80%	0.70%
Alcohol use disorders	1.80%	1.30%
Bipolar disorder	1.10%	0.50%
Eating disorders	1.10%	0.20%
Schizophrenia	0.40%	0.30%

Of note, data is limited to males and females, with no data or cognizance of clients who do not identify as male or female. Without looking at age stratification, the risk of CVD in AOD and/or depression could be mistakenly limited to the elderly, where CVD is known to be more prevalent.

Grey literature: The grey literature search further revealed that evidence of the concept of the WBV test for risk of bleeding and thrombosis has been known, predating the past century. Ten examples are provided in Table 6 as a snapshot of various applications identified in the literature [49].

**Table 6:** Known applications of whole blood viscosity. CVD = cardiovascular disease

	References	Application
1	[50,51]	Apheresis/CVD
2	[4,52]	Bleeding risk
3	[53,54]	Deep vein thrombosis
4	[55,56]	Dementia
5	[57,58]	Diabetes
6	[59,60]	Hypertension
7	[61,62]	Retinopathy
8	[63,64]	Sickle cell
9	[7,65]	Stress
10	[66,67]	Stroke

Discussion

The concept of the WBV test for risk of bleeding and thrombosis has been known for decades. It is acknowledged that most medical facilities lack the equipment to run the WBV test, with ultrasound imaging suggested as an alternative [44]. Yet, the eWBV algorithm method has also been known for some decades [7], and cognizance must be given to the affordances (i.e., accessibility and affordability) of ultrasound services that are still being improved upon [48]. Hence, the eWBV test has been of interest at least since 1981 [68], and 10 examples are provided here as a snapshot of various applications, including apheresis [50,51], bleeding risk [4,52], deep vein thrombosis [53,54], dementia [55,56], diabetes [57,58], hypertension [59,60], retinopathy [61,62], sickle cell disease [63,64], stress [7,65], and stroke [66,67]. In corroboration of the examples, many of the listed diseases impair blood viscosity [49].

Summary of Findings and Inferences From the Critical Appraisal

**Figure 2:**
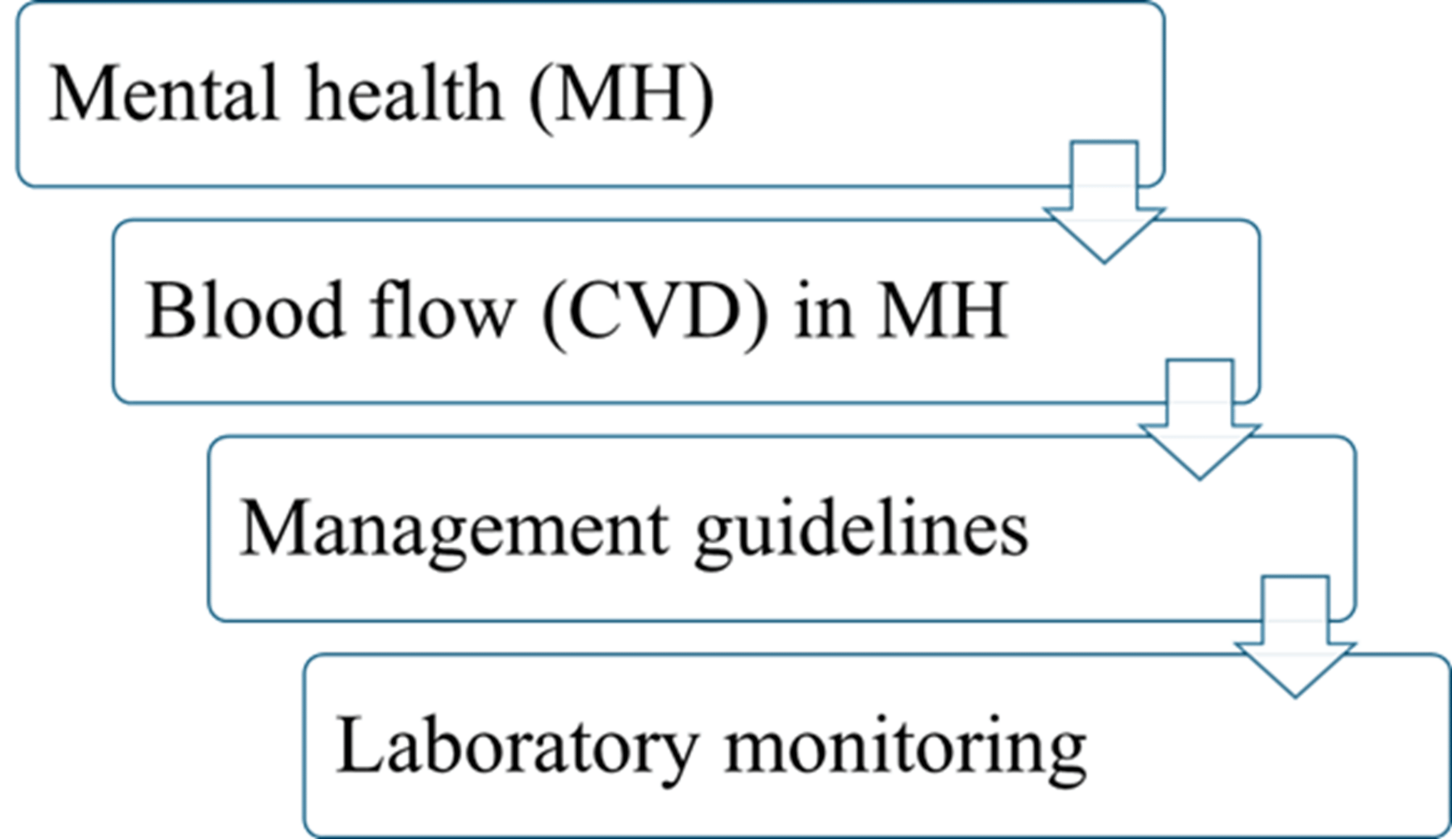
A graphical narrative of links between themes. CVD = cardiovascular disease

Figure 2 presents a graphical overview of how this narrative shifts between “mental health burden” and “blood flow (CVD complications) in mental health epidemiology,” to “management guidelines,” to “laboratory medicine (eWBV).”

Demography: There is data on age, gender, education, marital status, and occupational status. Results show that the prevalence of mental health in the Australian population is higher among younger adults aged below 50 years relative to those aged above 60 years. However, data on gender seems to exclude individuals who do not identify as female or male, i.e., the lesbian, gay, bisexual, transgender, queer, questioning, intersex, and asexual (LGBTQIA+) community. Therefore, further work is required to elucidate sociodemographic factors, especially age and sexual orientation.

Epidemiology of CVD: Evidence exists on the prevalence of mental health disorders (i.e., seven illnesses that constitute mental health categories listed in Table 2) as well as CVD in mental health. However, specifications on the prevalence of CVD in mental health clients are limited, indicated by the lack of such data on OWiD. Inference from the analysis of the results implies that CVD contributes to a higher mental health burden in Australia, but this requires empirical research.

Guidelines: What this study contributes is a proposal for a novel application of a known idea, whereby eWBV constitutes a repository of open-access benchmarked test protocols for pathology and evidence-based cardiovascular medicine outcome measures. This observation implies that clinical practice guidelines are not the focus of most researchers, and the inference is that the potential for advancing change may need to come from practitioners. With special focus on the nursing perspective, nurse practitioners could become more interested in research to advance knowledge regarding improvements in guidelines.

Pathology: There is plenty of literature, with no dispute on the usefulness or validity of eWBV. Yet, the test is unavailable or inaccessible in most clinical practice settings. Therefore, this study suggests that most medical facilities can measure WBV, but what is required is educating the healthcare practitioners on the eWBV algorithm. It is pertinent to emphasize that every medical facility may not have ultrasound equipment, but can access routine laboratory tests for hematocrit and liver function to ascertain blood viscosity at no further cost.

Gaps in Knowledge and Practice

Table 2 highlights the high quality of research in the literature. Especially, the evaluated literature on guideline and pathology themes received absolute affirmative responses from reviewers to all questions. Therefore, there are apparent gaps in knowledge and practice with regard to what is known about WBV versus the clinical practice.

Metabolic syndrome, CVD, and mental health disorders have a cause-and-effect relationship with each other. Between them are lifestyle behaviors as well as stress, making WBV applicable in stress management [7]. What is not clear is the adoption of empirical data from routine pathology tests in evidence-based monitoring of the management. The eWBV test could provide pathology EBLM to inform clinical decision-making regarding blood flow. It is readily available from routine tests, but it has yet to be utilized in clinical practice. The relationship between mental health and metabolic syndrome can be seen in people living with depression, where increased stress hormone levels cause hyperglycemia, hypertension, and obesity [69]. This cause-and-effect relationship is also described in the context of neuroticism, that is, emotional stability confers the correlation between mental health traits and CVD [70].

Mental health conditions and/or treatment of their side-effects can predispose individuals to CVD [71]. For instance, depression can complicate platelet hyperactivity and risk of thrombosis [69]. Hence, eWBV can aid in evaluating CVD management in depression. It is pertinent to bring to the fore the fact that blood flow dynamics and hemorheology have been a consideration in cardiovascular pathology for some decades [72], as well as in psychological stress management [7]. Therefore, the indication of high-quality research vis-à-vis knowledge about eWBV that is as yet not in clinical practice constitutes a gap in knowledge and practice. Perhaps, instead of the use of magnetic resonance imaging [73], this review suggests a laboratory method that is available to general practitioners and outpatient services.

In the CVD-depression nexus, socioeconomic variables, including age, educational status, gender, and unemployment, are demographic determinants [74], with financial burden constituting yet another demographic factor [75]. Although some demographic data are available on OWiD, this review reports a paucity of information, which calls for further research to bridge the gap. For instance, the need for empirical clinical research data on eWBV effectiveness in monitoring mental healthcare services.

Evidence for the Health Service

Tables 3-5 highlight the prevalence of mental health as well as age and gender differences, which are useful in health service management to appreciate the benefit for the EBM practice under discourse. It has been suggested that intervention studies in mental health should focus on areas for effective preventive management across diagnostic points to improve quality of life [76]. Mental health is among the leading causes of disability worldwide, as well as a major epidemiological factor in the pathogenesis of CVD and other chronic non-communicable diseases vis-à-vis metabolic syndrome. This fact underpins consideration of preventive medicine for CVD in mental healthcare [77]. Therefore, this review advances the diagnostic point of CVD, as evidenced by pathology, whereby monitoring for the effectiveness of preventive or therapeutic management may benefit from eWBV assessment.

Studies reported that while CVD mortality has decreased over the years due to improved diagnosis and treatment, the subpopulation with severe mental illness, contrastingly, lost years of life expectancy [78]. A supporting report highlighted that metabolic syndrome is the most prevalent cause of death in mentally ill people. Further, the need for metabolic syndrome control is emphasized, especially considering the lack of a multi-risk factor approach for CVD management in mental health [79]. Therefore, the significance of this report in the potential use of available pathology EBM in CVD management.

Evidence for Clinical Nursing Practice Including Mental Health

It is pertinent to mention that the mechanisms of some dietary interventions in mental healthcare have been established to include influences on prostaglandins and thromboxane, which are important in platelet function [80,81], potentially leading to improving blood flow [82-84]. However, investigations to adopt and advance this in clinical practice have yet to occur. Further, another study with recourse to Australia showed “fundamental absence of adequate integrative models of care within mental health services to enable the optimal nursing care of people with combined mental health and substance use disorders” [85]; hence, further research is suggested to assess, for instance, factors that could influence nursing roles in integrative care. Yet, in the context of integrative or the perspective of nursing practice in mental health, several studies abound, but are not focused on cardiovascular care [86,87]. Hence, this review contributes to the discourse by focusing on evidence-based cardiovascular medicine.

Health Economics Implications: Cost of eWBV Measurement

Table 6 outlines some examples of CVD where eWBV has been applicable, albeit in a research setting. It is pertinent to clarify that the eWBV measurement has up to three options. The first is using the algorithm formula available from the Atherosclerosis Risk in Communities (ARIC) article [88]. This is achievable by inputting the formula in an Excel document that enables offline usage. A second option, which is also offline usage, is the user-friendly extrapolation chart, which is also available for print [8]. A third option is by using the online algorithm, i.e., digitally (https://gmrdo.org/check-blood-viscosity-thinness-thickness/), which merely requires inputting the results of hematocrit and serum protein results. With the digital option comes flexibility for clients, doctors, and nurses in being able to generate the eWBV results on their computers or phones, which facilitates discussion.

Strengths and limitations of the study

A systematic reviews often contrate on a single domain or precise topic, whereas a scoping review method is broader and useful when a phenomenon of interest may be unclear or cannot be validly addressed by a precise research question [89], such as consideration of associated influencing epidemiological or practice factors.

Further, an integrative review is yet another approach that addresses multiple research questions [90,91]. This study, though focused on EBLM, has given recourse to the concepts of epidemiology and guidelines. Therefore, one strength of this is the in-depth, rigorous, and concise review, giving cognizance to a broader scope than traditional reviews.

The second strength of this review is that the quantitative data are based on an empirical and verifiable database, OWiD, which has been used as a resource in previous studies [92,93]. It is one of the emerging platforms for empirical secondary data collection and analysis [94]. Perhaps, it is noteworthy that in-depth and rigorous scoping and/or systematic reviews are required to go beyond peer-reviewed journal articles [95,96] and include evidence from other relevant sources [97]. Indeed, scoping reviews are “more likely to use nonindexed sources” [98].

In high-quality critical appraisal, identifying appropriate criteria and checklists and selecting an appropriate set of criteria and checklists are two different steps. Further, critical appraisal is expected to aim at focusing on the discriminated problem, educational advancement, adoptability, or relevance of the research [99]. In this study, the critical appraisal is limited to evaluating the adoptability and relevance of the reports. However, considering that the novelty of research is defined by the new knowledge created, the expectation of systematic review is to define key concepts and identify gaps in each field [91,100]. Therefore, this work has strength.

## Conclusions

This study provides evidence that despite the high quality of published research, there are gaps in knowledge and practice regarding the adoption or translation of eWBV in clinical practice. It justifies a proposal for a novel application of a known idea, whereby eWBV constitutes a pathology test protocol for EBLM to monitor blood flow as a cardiovascular outcome measurement. Thus, there is a need for at least clinical audit research involving review of laboratory data in conjunction with clients’ treatment histories, with a view to using real-life medical records to improve EBLM services in mental healthcare.
